# Genetic structure of American bullfrog populations in Brazil

**DOI:** 10.1038/s41598-022-13870-2

**Published:** 2022-06-15

**Authors:** Gabriel Jorgewich-Cohen, Luís Felipe Toledo, Taran Grant

**Affiliations:** 1 Instituto de Biociências, Universidade de São Paulo, Cidade Universitária, Rua Do Matão, 101, São Paulo, SP CEP 05508-090 Brazil; 2 Paläontologisches Institut Und Museum, Universität Zürich, Zürich, Switzerland; 3 Laboratório de História Natural de Anfíbios Brasileiros (LaHNAB), Departamento de Biologia Animal, Universidade Estadual de Campinas, Campinas, SP 13083-862 Brazil

**Keywords:** Population genetics, Ecological genetics, Molecular ecology

## Abstract

Non-native species are a major problem affecting numerous biomes around the globe. Information on their population genetics is crucial for understanding their invasion history and dynamics. We evaluated the population structure of the non-native American bullfrog, *Aquarana catesbeiana*, in Brazil on the basis of 324 samples collected from feral and captive groups at 38 sites in seven of the nine states where feral populations occur. We genotyped all samples using previously developed, highly polymorphic microsatellite loci and performed a discriminant analysis of principal components together with Jost’s D index to quantify pairwise differentiation between populations. We then amplified 1,047 base pairs of the mitochondrial cytochrome *b* (cytb) gene from the most divergent samples from each genetic population and calculated their pairwise differences. Both the microsatellite and cytb data indicated that bullfrogs comprise two populations. Population grouping 1 is widespread and possesses two cytb haplotypes. Population grouping 2 is restricted to only one state and possesses only one of the haplotypes from Population grouping 1. We show that there were two imports of bullfrogs to Brazil and that there is low genetic exchange between population groupings. Also, we find that there is no genetic divergence among feral and captive populations suggesting continuous releases. The limited genetic variability present in the country is associated to the small number of introductions and founders. Feral bullfrogs are highly associated to leaks from farms, and control measures should focus on preventing escapes using other resources than genetics, as feral and captive populations do not differ.

## Introduction

The introduction of non-native species as a result of human actions is one of the major causes of wildlife threat and extinction around the world^1,2^ and can result in great biological and economic losses^3–5^. Precautionary policies to prevent new introductions and dissemination of already introduced populations are essential^6^, as are efforts to control and eliminate established non-native species^7,8^. The effort and cost of controlling invasive species can be prohibitive, especially for species that are distributed over large areas and are continually reintroduced^9^, like the north American bullfrog (hereafter bullfrog), *Aquarana catesbeiana*^10^, in Brazil.

Native to eastern north America, bullfrogs are, relative to most anurans, large and voracious predators, exceeding 150 mm in adult body length^11,12^ and consuming a broad diversity of animal taxa, including small vertebrates^13–18^. Egg clutches comprise as many as 20,000 eggs and frogs have a fast growth rate, reaching sexual maturity within 1 year of metamorphosis^19^.

Although the specific responses of native fauna to introduced bullfrogs remain poorly understood, many studies have examined the bullfrog invasion around the world^20,21^ and have found that bullfrogs can have a significant impact on native fauna^22^: Kupferberg^23^ reported larvae competition among native species in consequence of phytoplankton change in pools invaded by bullfrogs. The voracity and generalist diet of introduced bullfrogs have led to concerns about possible decrease of native species’ populations^13,15,18,24^. Similarly, given that bullfrogs are resistant to chytridiomycosis caused by *Batrachochytrium dendrobatidis* (*Bd*) infection^25–27^, one of the primary causes of precipitous amphibian population declines, the occurrence of *Bd* in a large proportion of bullfrogs in Brazilian frog farms^28–31^ suggests that feral bullfrogs might be an important disease vector. For example, bullfrog presence has been found to be a positive predictor of both *Bd* prevalence and *Bd* load in the north American frog *Rana boylii*^32^. In Brazil, the most direct evidence of impacts on native frogs is provided by experimental studies that provided evidence of a change in the vocal behavior of native males, that increased their vocal frequency in response to bullfrog vocalizations^33,34^, although this represents niche overlap and does not necessarily impact species diversity. A recent report indicates that bullfrogs in Brazil seem to have little influence on native amphibian population dynamics^35^, but a study focused on its known and potential impacts indicates that the presence of bullfrogs can cause changes in activity and habitat use preferences of native species^36^.

Although there is very limited knowledge about the possible impacts of bullfrogs over native species in Brazil, this species is widespread in the south and southeast regions of the country, with limited distribution in the northeast and north^37,38^. There are over 150 known breeding facilities in the country, that produce around 400 tons/year, moving around 1.9 million USD^38^. According to published accounts, bullfrogs were first imported to Brazil to produce meat for human consumption in 1935 by a Canadian technician named Tom Cyrril Harrison who brought either 300 individuals^39^ or pairs^40^ from an unknown locality to a Brazilian government breeding facility in the city of Rio de Janeiro^41,42^. Hundreds of tadpoles were subsequently sent to new breeding facilities around the country in an agricultural program encouraged by federal and state governments^41,42^. The state-based characteristics of this governmental program facilitated the appearance of feral bullfrog populations in different political regions of the country, as today feral bullfrogs are widespread in the south and southeast regions of Brazil, with additional localities in the central-west, northeast, and north^41^. Escape and release of bullfrogs from farms are spatially correlated with feral bullfrog populations^43–45^, being the most likely cause considering the disconnected distribution of these populations in Brazil, although the dispersive potential of bullfrogs is not well documented. Although advances in technology and increased market demand have enabled bullfrog farming to expand greatly over the last 10–15 years^46^, many of the earlier frog farms failed in the 1990s^38^, resulting in massive releases of bullfrogs when businesses closed (Valdir Alves, owner of Pedrinhas bullfrog farm, personal communication). In the 1970s, an additional 20 pairs of adult bullfrogs were imported to Brazil^28^ from the University of Michigan to São José do Rio Preto in São Paulo state by Luiz Dino Vizotto (C. M. Ferreira, personal communication). Little information about these individuals is available, and the outcome of this introduction is not confirmed nor discussed in the literature. The same is true for small-scale occasional introductions supported by individual breeders, without any official registers, such as a supposed bullfrog batch introduced from Mexico in the early 2000’s^38^.

If all the introduction events were successful, different lineages in the country might be going through an introgressive hybridization process due to the common practice among farmers of purchasing breeding stock from multiple farms in different states with the goal of increasing the genetic diversity of the frogs bred in their facilities (Romar’s bullfrog farm owner, personal communication)^38^. If only one event was successful, followed by strong selective pressures of breeding facilities, the genetic diversity of bullfrog populations in Brazil should be quite limited. The limited information about introduction history of the bullfrog in Brazil raises the possibility of different genetic scenarios. At least one population was successfully introduced in the country, and other lineages could have also contributed genetically to the introduced populations.

Information on introduced bullfrog population genetics is crucial to understand invasion history, structure and dynamics of gene pool exchange between populations^47,48^, and can be helpful to develop management and control programs^49,50^. Efforts to understand the genetic structure of introduced bullfrog populations have been undertaken in several regions, such as Europe^51^, China^43^ and western USA^48,52^. Unlike the invasion history in Brazil, Europe had several events of introduction from different origins^51^, and the western region of the USA is continually flooded with individuals from the native range^52^. China also had different events of introduction, although little genetic diversity was found^43^. These examples can serve as a baseline for comparison in new genetic research, and give indications about the invasiveness of a non-native population.

Because multiple introduction events can create introduced populations that present more diverse gene pools^52^, we expect introduced bullfrog population in Brazil to be less diverse than other non-native populations previously studied in different regions of the globe, as fewer introduction events were reported in Brazil in relation to other areas. Considering that more unreported introduction events could have happened, our main goal in this study was to assess the genetic structure of Brazilian bullfrog populations and possible gene flow between them. To achieve our goal, we focused on four main questions: (1) Are captive and feral populations genetically different? (2) Does bullfrog genetic structure corroborate the hypothesis that there were two or more introduction events in Brazil? (3) Is there gene flow between populations? (4) Do Brazilian bullfrog populations present lower genetic diversity than non-native populations from other regions?

## Materials and methods

**Figure 1:**
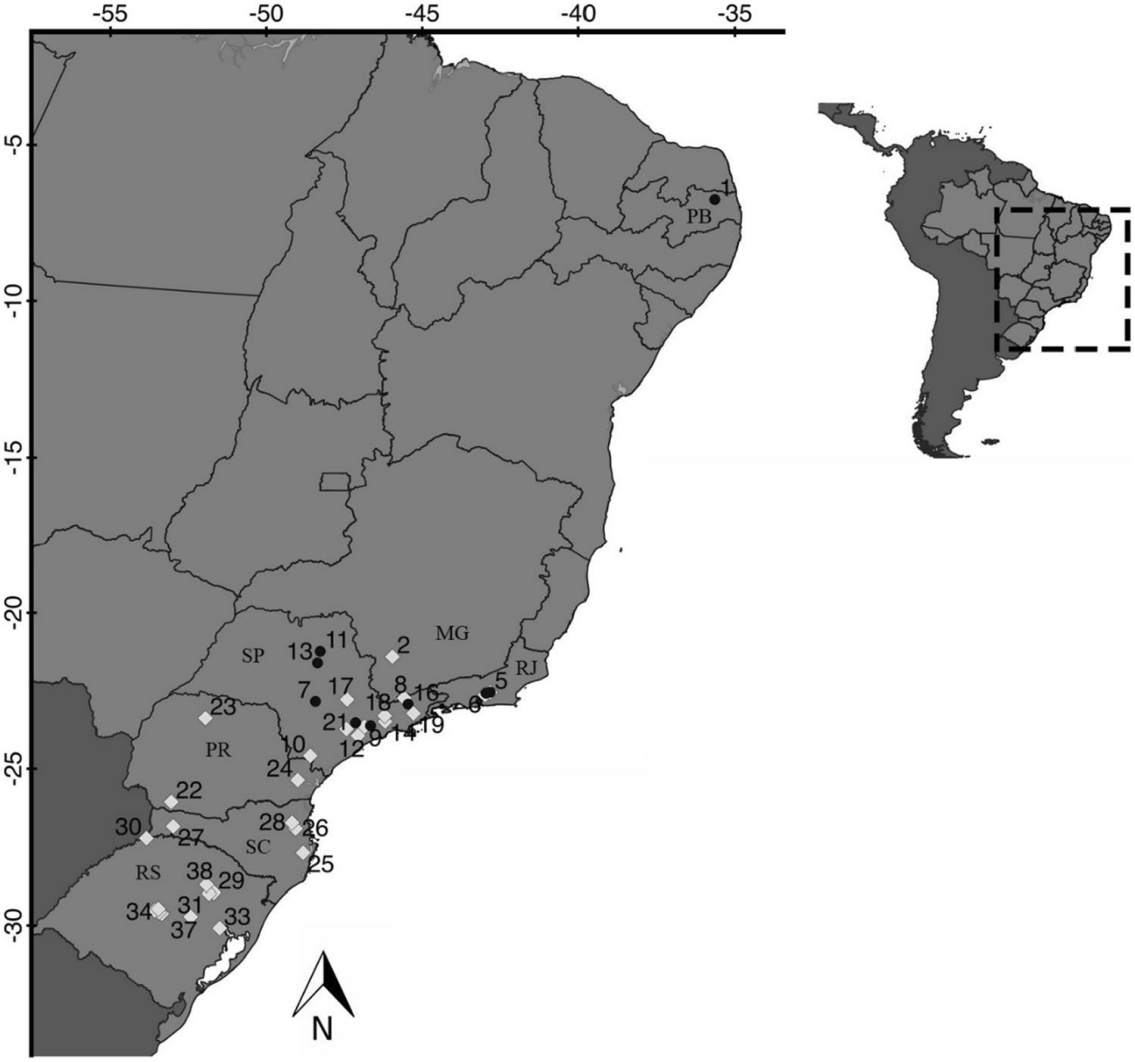
Sampling locations of bullfrogs in Brazil. Include captive (circle) and feral (diamond) specimens. Sample size is reported in parentheses. 1. Bananeiras, Universidade Federal de Paraíba frog farm (22); 2. Alfenas (21); 3. Magé, Vila Pedrinhas frog farm (16); 4. Cachoeira de Macacu (10); 5. Cachoeira de Macacu, Andre’s frog farm (16); 6. Guapimirim, Romar frog farm (25); 7. Botucatu, Universidade Estadual de São Paulo (UNESP) frog farm (20); 8. Campos do Jordão (4); 9. Embu das Artes (4); 10. Iporanga (1); 11. Jaboticabal, UNESP frog farm (4); 12. Juquitiba (1); 13. Matão, Ranamat frog farm (10); 14. Mogi das Cruzes (5); 15. Piedade (9); 16. Pindamonhangaba, Vale sereno frog farm (7) ; 17. Santa Barbara D’oeste, Santa Rosa frog farm (3); 18. Santa Isabel, Santa Clara frog farm (4); 19. São Luiz do Paraitinga (3); 20. São Paulo, Santa fé frog farm (3); 21. São Roque, Ranaville (9); 22. Francisco Beltrão (15); 23. Maringá (16); 24. Quatro Barras (5); 25. Águas Mornas (1); 26. Blumenau (2); 27. Pinhalzinho (1); 28. Pomerode (5); 29. Cotipora (1); 30. Derrubadas (7); 31. Dois Lageados (1); 32. Dona Francisca (1); 33. Eldorado do Sul (2); 34. Faxinal do Soturno (26); 35. Ivora (2); 36. Nova Palma (16); 37. Santa Cruz do Sul (25); 38. Serafina Correa (1). States where bullfrog samples are represented with their initials: Paraíba (PB), Rio de Janeiro (RJ), Minas Gerais (MG), São Paulo (SP), Paraná (PR), Santa Catarina (SC), and Rio Grande do Sul (RS).

We obtained 324 bullfrog skin, liver, and/or muscle tissue samples, of which 128 were purchased from 11 farms and 194 were collected from feral specimens or sampled from museum or private collections (Fig. 1; Table SI). Specimen collection was conducted in accordance to the guidelines and authorized by the Instituto Chico Mendes de Conservação da Biodiversidade (SISBio 56772-1). The sampling methodology also followed the ARRIVE guidelines^53^. Farm animals were not sacrificed for this research. Tissues were stored in 99% ethanol and kept at − 20 °C until DNA extraction, which we performed with the DNeasy Blood and Tissue extraction kit (Qiagen, Valencia, CA, USA) following the manufacture’s guidelines.

### Microsatellite analyses

We amplified seven nuclear microsatellite loci using the library developed by Austin et al.^54^. Multiplex PCR was performed on a Veriti™ thermal cycler (Applied Biosystems) with a thermal profile consisting of 95 °C for 7 min followed by 10 cycles of 95 °C for 30 s, touchdown from 62 to 57 °C for 45 s, and 72 °C for 30 s, followed by 30 cycles of 95 °C for 30 s, 50 °C for 30 s, and 72 °C for 30 s, and final extension at 72 °C for 7 min. The reaction mix, with a total volume of 10 µl, contained (1.0 µl) buffer, (0.5 µl 2 mM) dNTPs (0.5 µl) fluorescent dye (VIC for RcatJ11 and RcatJ44b; NED for RcatJ21 and RcatJ41; PET for RcatJ54 and Rcat3-2b or 6-FAM for RcatJ8; applied biosystems), (0.5 µl, 5 µM) of mixed forward and reverse primers, (0.125 µl) Taq polymerase, (3.375 µl) distilled deionized water, and (3.0 µl) template DNA. Later, we diluted the PCR products to a proportion of 1:4 and submitted them to sequencing by a third party, not changing service provider to avoid bias due to calibration differences. We scored results using Gene Marker v. 2.6.3 (SoftGenetics) and tested for the presence of null alleles, allele dropout, and stuttering using Microchecker^55^. We also genotyped 91 samples (24.1%) a second time to evaluate the percentage of homozygotes that were actually null alleles. We included control samples in each procedure.

**Figure 2:**
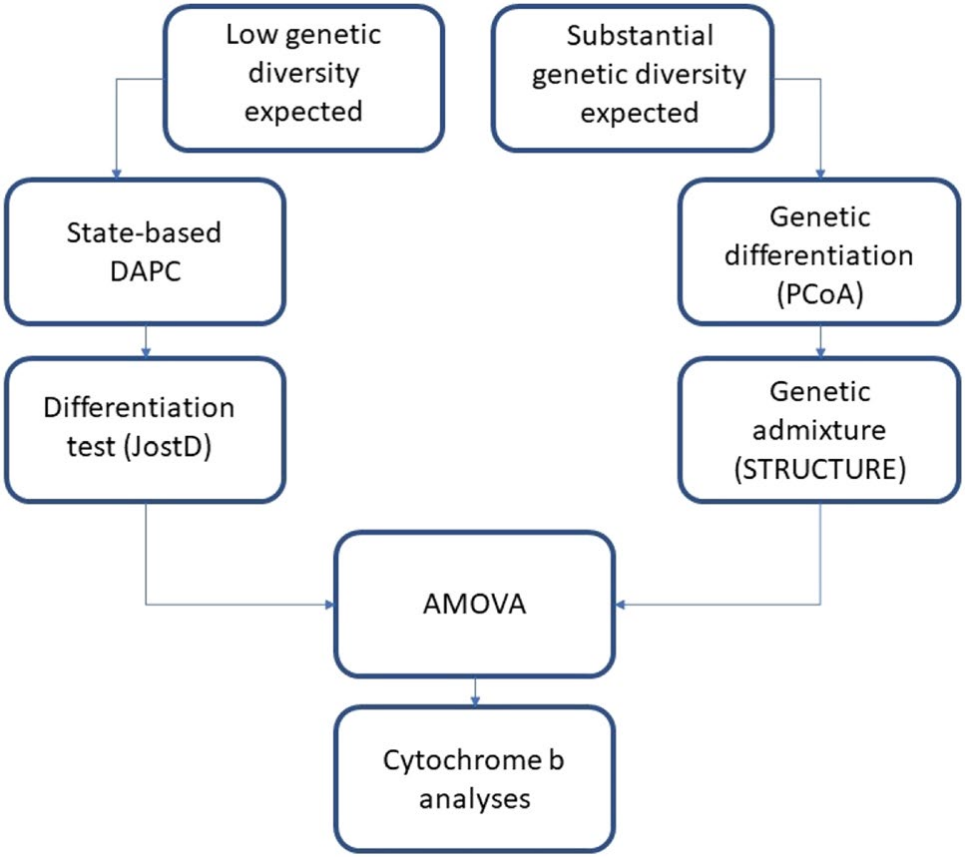
Flowchart of decision making for microsatellite analyses explains the decision-making pathway considering possible different scenarios and analyses biases.

Given that published sources report a total of 340 or 640 bullfrogs being imported to Brazil (300–600 in 1935 and another 40 in the 1970s^28,39,40^, we anticipated two possible scenarios: (1) extremely low genetic divergence and diversity among bullfrog populations in Brazil. This could have been caused by limited genetic diversity in the founding event of Brazilian populations, together with strong selective pressure in breeding facilities; 2. Increased genetic diversity caused by founding populations that were prevenient from different native populations. Considering that each scenario would require different analytical methods, we chose for assessing the genetic structure using two different analytical toolkits. A flowchart with the analytical decision-making is available in Fig. 2.

For scenario 1, we performed a discriminant analysis of principal components (DAPC)^56^ in the adegenet R package^57^ to assess current genetic structure. This analysis includes all bullfrog samples in the country grouped by state, in recognition of the state-based government programs regulating bullfrog farming. To determine if there is any genetic differentiation between groups defined by feral or captive origin from the same locations—which could be used to inform law enforcement in case of releases—we performed one DAPC only for states in which both groups were well sampled (Rio de Janeiro and São Paulo).

We used Jost’s D index^58^ to quantify pairwise genetic differentiation among state populations, which was calculated with the mmod R package^59^. Next, we tested the significance of differentiation between pairs of populations using the DEMEtics R package^60^ with 10,000 permutations. We applied the Benjamini–Hochberg Procedure^61^ to control for false discovery rates (FDR) and avoid type I error^62^ using the *p.adjust* function from stats package^63^. We used the gstudio package^64^ to calculate the following diversity indices for each genetic population: expected (He) and observed (Ho) heterozygosity, number of alleles (A) per locus, effective number of alleles (Ae) per locus, and size-corrected Wright's inbreeding coefficient (Fis). Through a permutation procedure with 100 batches of 1,000 iterations, we checked for Hardy–Weinberg equilibrium (HWE) and linkage disequilibrium (LD) in each state population by performing a probability test in Genepop ver. 3.4^65^. To avoid type I error, P values were corrected following the Benjamini–Hochberg Procedure.

Considering scenario two, we performed a principal coordinate analysis (PCoA) to visualize genetic differentiation using the R package Adegenet^57^. We estimated admixture using STRUCTURE software^66^. This analysis was performed using nine runs for every K (from K = 1 to K = 9) using state populations in the locprior model and a burn-in period of 10.000 steps and 20.000 Markov chain Monte Carlo (MCMC) repetitions. The best fitted K number of populations was calculated using STRUCTURE HARVESTER^67^, and interpreted through the Evanno method^68^.

We also calculated the differentiation among and within state populations and feral and captive populations through an analysis of molecular variance (AMOVA). We used Arlequin software^69^ to perform these analyses.

### Cytochrome b analyses

On the basis of the microsatellite results, we selected the 18 most divergent samples from each genetic population (Table 1) and amplified a 1047 basepair (bp) segment of the mitochondrial cytochrome *b* gene (cytb). We used a combination of the primers MVZ15L^70^ and cyt-bAR-H^71^ and a thermal profile for polymerase chain reaction that consisted of 95 °C for 10 min, followed by 45 cycles at 95 °C for 30 s, 50 °C for 40 s, and 72 °C for 40 s, with a final extension step at 72 °C for 5 min. The reaction mix, with a total volume of 25 µl, contained (0.15 µl) Go Taq G2 Flexi DNA Polymerase (Promega corporation), (2.5 µl) Go Taq flexi Buffer, (1.0 µl, 2 mM) dNTPs, (2.0 µl, 25 mM) MgCl2, 1.0 µl of each primer (10 pM), (15.35 µl) distilled deionized water, and (2.0 µl) template DNA. PCR amplification products were cleaned using Agencourt AMPure XP DNA Purification and Cleanup kit (Beckman Coulter Genomics, Brea, CA, USA), and they were sequenced by a third-party using fluorescent-dye labelled terminators (ABI Prism Big Dye Terminators v. 1.1 cycle sequencing kits; Applied Biosystems, Foster City, CA, USA) with an ABI 3730XL (Applied Biosystems, Foster City, CA, USA). Sequences are available on GenBank (MT668579 and MT668580).

**Table 1:** Samples selected for the mitochondrial cytochrome b locus sequencing.

*n*	Locality (Municipality, State)	Population
1	Derrubadas, Rio Grande do Sul	1
1	Quatro Barras, Paraná	1
1	São Luiz Paraitinga, São Paulo	1
3	Jaboticabal, São Paulo	1
1	Magé, Rio de Janeiro	1
1	Guapimirim, Rio de Janeiro	1
1	Francisco Beltrão, Paraná	1
1	Faxinal Soturno, Rio Grande do Sul	1
8	Alfenas, Minas Gerais	2

We used Geneious ver. 10.2.3^72^ for sequence editing and contig formation of the cytb sequences based on the chromatograms obtained from the automated sequencer. All samples were sequenced in both directions to check for potential errors. The sequences were aligned with the MAFFT^73^ plugin in Geneious 10.2.3 (Biomatters) with the G-INS-I strategy. We trimmed the sequence alignment to a length of 937 bp. We used Arlequin 3.5^69^ for most genetic analysis. We calculated the haplotype diversity (Hd) and nucleotide diversity (π) indices and report values ± SD for each genetic population. We also calculated the pairwise differences between the introduced populations using the θST index and evaluated its significance by performing 10,000 permutations. Using the *p.adjust* function from stats package^63^, we performed the Benjamini–Hochberg Procedure^61^ to control for FDR.

## Results

**Figure 3:**
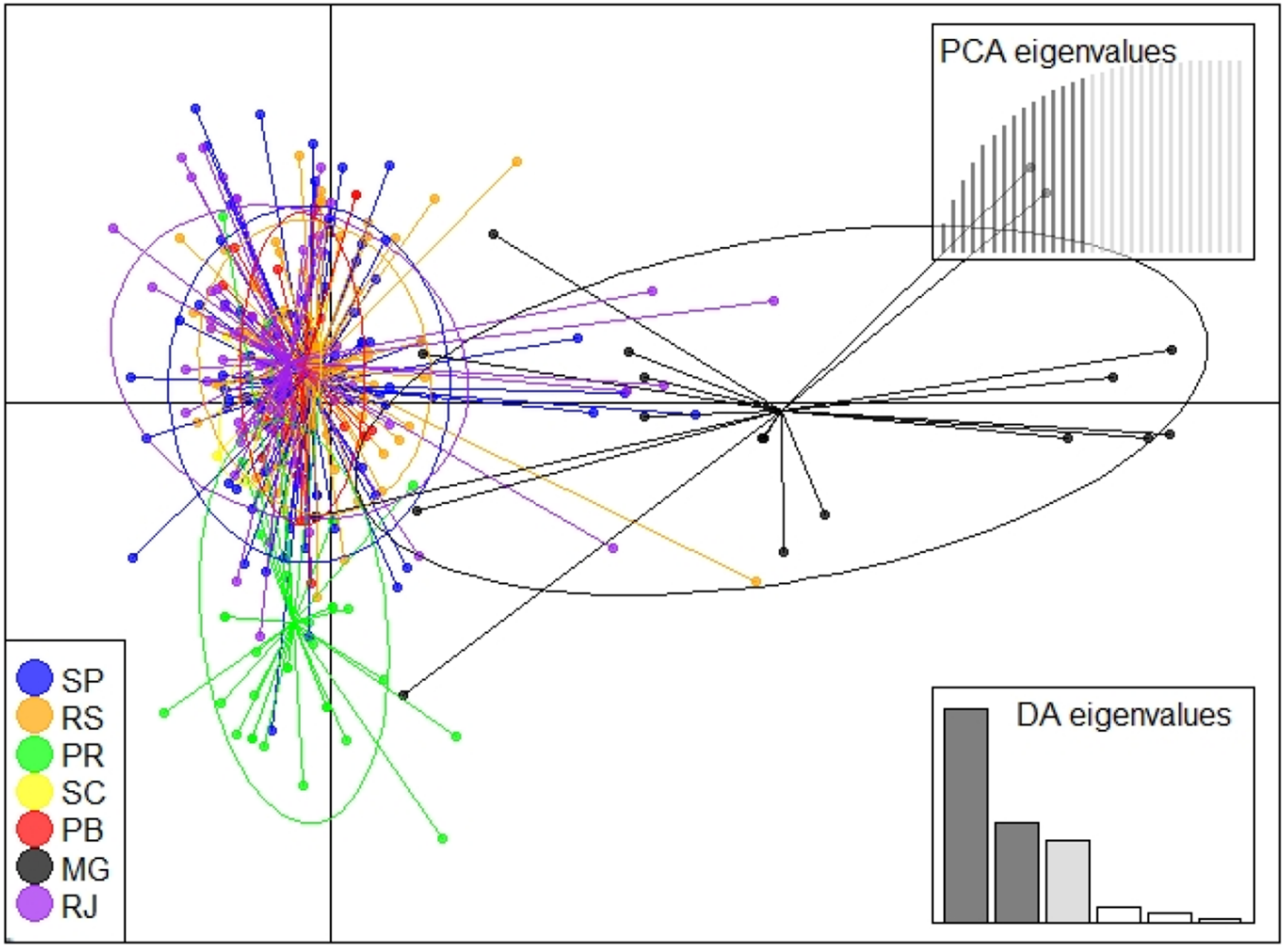
Discriminant analysis of principal components of bullfrog populations in Brazil. Acronyms represent different states: Paraíba (PB), Minas Gerais (MG), Rio de Janeiro (RJ), São Paulo (SP), Parana (PR), Santa Catarina (SC), and Rio Grande do Sul (RS). Metapopulations were organized by States due to the State-based production system implemented in the country.

The presence of null alleles was indicated for all loci, whereas allele dropout and stuttering measures were not significant. Genotyping error was estimated to be less than 2%. The state-based total-data DAPC (Fig. 3) showed extensive overlap between most sampling units (defined as Population grouping 1) except individuals from Minas Gerais (Population grouping 2).

Pairwise multilocus Jost’s D between Population grouping 1 and Population grouping 2 was significant even after FDR correction (Jost’s D = 0.25638, *P* = 0.001). Most pairwise comparisons indicated significant LD (Table SII) and deviation from HWE (Table 2), even after FDR correction. The mean number of alleles per locus (A) within each population was 4.28–9.57, while the effective number of alleles was only 2.86–4.05 due to the strong dominance of one or two alleles in most loci (dominant alleles accounted for 20–50% of the total at each locus). The observed heterozygosity did not match expected heterozygosity and both population groupings showed positive inbreeding values (Table 2).

**Table 2:** Characteristics of bullfrog populations in Brazil.

Group	Heterozygosity	Diversity				Hardy–Weinberg probability test *P* values						
	He	Ho	Fis	A	Ae	RcatJ11	RcatJ21	RcatJ54	RcatJ8	RcatJ44b	RcatJ41	Rcat3-2b
1	0.73	0.49	0.32	9.57	4.05	0.07	**0.04**	**0**	**0**	**0**	**0**	0.09
2	0.62	0.45	0.29	4.28	2.86	0.67	0.64	**0**	0.44	**0**	**0.01**	0.52

*He* Expected heterozygosity, *Ho* Observed heterozygosity, *Fis* Size corrected Wright's inbreeding coefficient, *A* Number of alleles per locus, *Ae* Effective number of alleles per locus. *P* values that are still out of Hardy–Weinberg equilibrium after False Discovery Rate corrections appear in bold.

**Figure 4:**
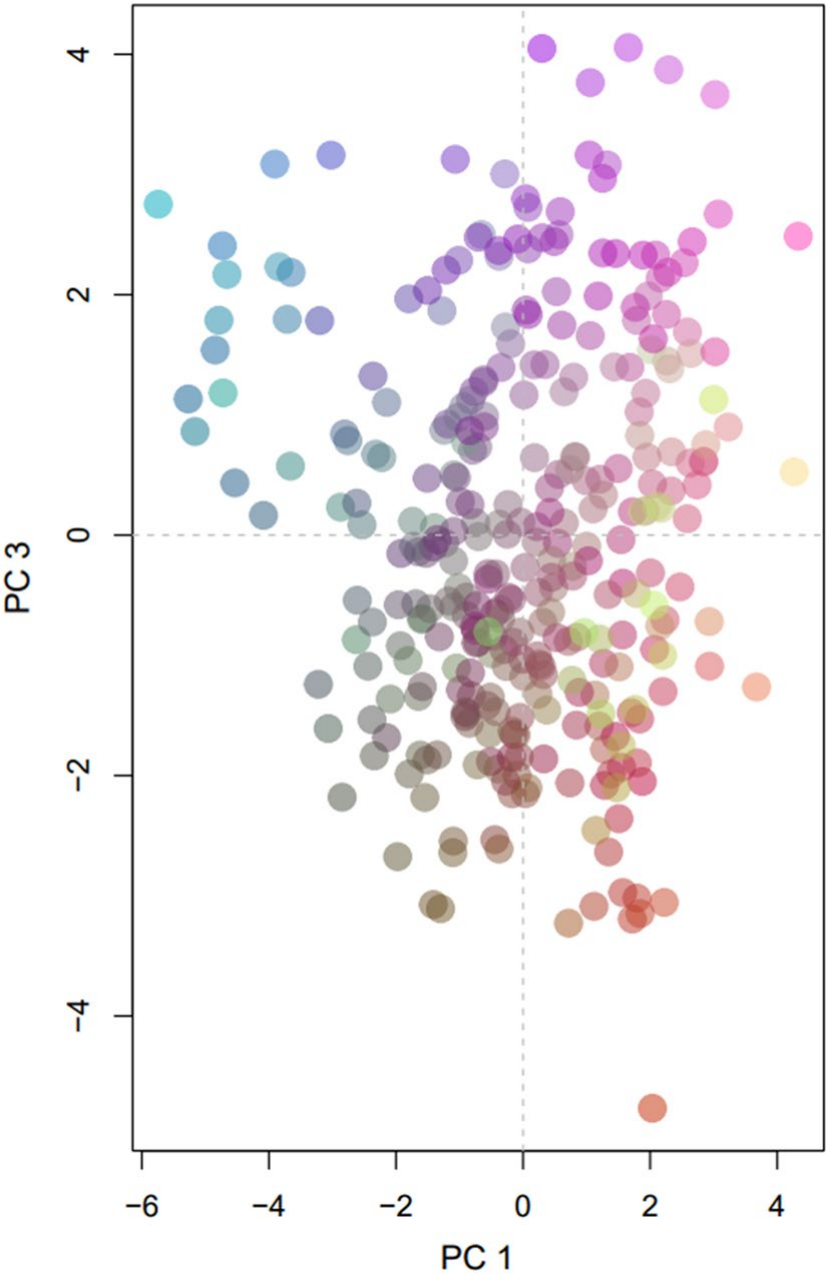
Principal coordinate analysis (PCoA) of bullfrog populations in Brazil used to visualize genetic differentiation among specimens from different sampling locations. Color gradient represents genetic variation among samples across PCs. Similar colors represent similar genetic structure.

**Figure 5:**
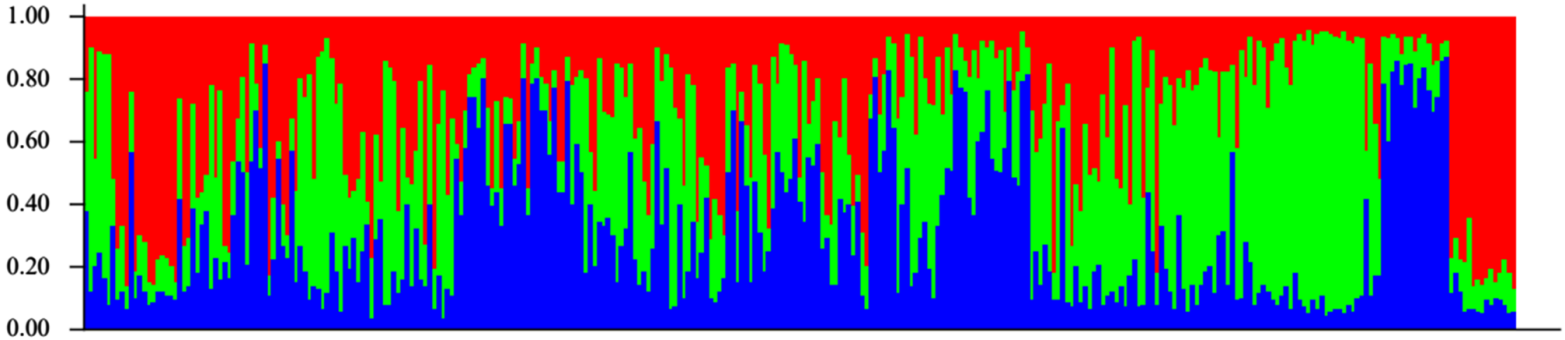
Admixture of bullfrog populations in Brazil Proportions of admixture (K = 3) among bullfrog specimens from captive and feral populations.

The PCoA analysis did not present any patterns of genetic differentiation (Fig. 4. Conversely, the clustering analysis performed by STRUCTURE found K = 3 to the best fit for genetic populations (Fig. 5), supported by the Evanno method (Supplemental material, Fig. SII). The state-based AMOVA (Table 3) indicated 77.49% of the genetic variation should be within individuals, and only 2.98% among groups (states). Similar results were found for the AMOVA between feral and captive specimens (84.39% and 0.07%, respectively). The DAPC that tested the differentiation between all feral and captive groups (Supplemental material, Fig. S1) presented almost complete overlap of sample units, supporting the results found in the AMOVA and leading to the conclusion that feral and captive animals are not significantly different.

**Table 3:** analysis of molecular variance (AMOVA) among and within state-based populations.

Source of variation	d.f	Sum of squares	Variance components	Percentage of variation
Among groups	5	60.25	0.04 Va	2.98
Among populations within groups	27	130.94	0.18 Vb	11.3
Among individuals within populations	292	457.45	0.13 Vc	8.22
Within individuals	325	420	1.29 Vd	77.49
Total	649	1068.65	1.66	100

We identified only two cytb haplotypes in Population grouping 1 haplotypes (Haplotypes A and B) that differ from each other in 14 base pairs (π = 0.005324 ± 0.003); Haplotype A was more frequent than Haplotype B, occurring in 80% of specimens (Hd = 0.3556 ± 0.159). Population grouping 2 contains Haplotype B exclusively. The population differentiation test between the two population groupings was significant after FDR correction (pairwise θST = 0.75535, P = 0.00147).

## Discussion

All bullfrogs in Brazil descend from a relatively small number of founders introduced at most 85 years ago, in at least two different events^28,39,40^. The introduction history of this species resulted in high rates of homozygosity, high inbreeding coefficient values, low allelic richness, and consequent overestimations of LD and HW disequilibrium. These characteristics are predictable, as they are associated with the founder effect and inbreeding in these populations following their importation to Brazil and subsequent intensive reproduction and presumed artificial selection. Populations with low genetic variation, as expected in cases of non-native populations with few founders or populations that have undergone major reductions^73^, present special conditions that require appropriate methods. As such, we used DAPC to analyze genetic structure because it makes no assumptions about HWE, LD, or gene flow, like more commonly used analyses such as STRUCTURE^66^.

Similarly, although null alleles are known to inflate measures of genetic differentiation and create false homozygotes^74,75^, the common practice of discarding loci inferred to possess null alleles [e.g.^50,77,78^ is contraindicated in cases such as the introduced bullfrog in Brazil. Specifically, Microchecker indicates the presence of null alleles on the basis of excess homozygotes being evenly distributed across homozygote classes^55^, which is precisely the situation expected to occur in recent invasions subjected to high levels of inbreeding [e.g.,^9^. The indication of null alleles in all genes reinforces this point, as all genes underwent the same biological process and none of them are in HWE. Nevertheless, the double genotyping of almost a quarter of all microsatellite data showed that the presence of null alleles is not significant. Thus, we included all loci in our analyses.

Considering that other non-reported introductions could have happened, increasing the gene pool and contradicting our first methodological toolkit assumptions, we reanalyzed our dataset with a more traditional approach. This included PCoA and STRUCTURE, to visualize genetic differentiation and admixture, respectively; and AMOVA, to teste differentiation among populations.

Both methodological toolkits presented similar results, with low differentiation among population groupings. Our state-based analysis of microsatellite data corroborates published accounts that there have been only two importations of bullfrogs to Brazil, a well-known importation of 300–600 individuals in 1935 and a lesser-known importation of 20 pairs in the 1970s^28^. Specifically, although we observed that samples from most areas overlap extensively by occupying the same multivariate space in the DAPC (Population grouping 1), samples from Minas Gerais did not (Population grouping 2). Similarly, the best number of Ks indicated for the STRUCTURE analysis was three. Although there was a difference in the interpreted number of populations between the two used approaches, it could have happened due to biases in both of them. The DAPC approach is subjected to a certain amount of subjectiveness, as different populations are interpreted by their distribution in the multivariate space. In this case, the population from Parana, for instance, has a distribution that does not completely overlap with the rest of samples named here as Population grouping 1. This could be the third K represented in the STRUCTURE approach. At the same time, as mentioned before, the assumptions about HWE, LD, or gene flow in the STRUCTURE analysis could also be responsible for inflating the real number of Ks.

The results of the cytb analysis corroborate the DAPC results and further suggest that either founders of Population grouping 1 contained both mtDNA haplotypes, or some degree of introgression of the haplotype from Minas Gerais (Population grouping 2) into Population grouping 1 has occurred but not the other way around. This might be correlated with the decrease of frog farmers in the state of Minas Gerais, previously known as one of the biggest producers in the country. Farmers from other states, like São Paulo where some haplotypes from population grouping 2 were found, made efforts to enhance the genetic diversity of their breeding population by importing animals from other states while breeders from Minas Gerais closed or reduced their facilities, preventing Population grouping 2 from receiving migrants from Population grouping 1. We therefore conclude that populations from the same genetic units are probably exchanging migrants, since there is no signal of genetic differentiation between them, what agrees with the information that farmers keep exchanging animals. This practice is unlikely to be effective, given the low genetic variation of bullfrogs in Brazil, and might also have contributed to preventing the genetic differentiation of introduced populations. That is, by exchanging "migrants" with each other and replenishing feral populations with constant leaks^44^, breeding facilities effectively maintain bullfrog populations across the country in an infinite island model. This process was documented in a recent work^32^, where authors investigated the historical and present bullfrog trade among Brazilian states. In their work, the states of São Paulo, Rio de Janeiro and Minas Gerais seem to be central to this market. They did not contrast the trade of living bullfrogs from the meat trade, what might explain why Minas Gerais still has differentiated genetic traits even though it is deeply entangled in the commercial net.

The lack of differentiation among populations prevents the use of genetic resources to diagnose and control leaks from breeding facilities. As shown in both DAPC and AMOVA, captive and feral specimens do not differentiate. This result can be explained not only by biological processes, like the low number of founders and intense inbreeding following importation, but also by the common practice among farm managers of purchasing breeding stock from multiple farms in different states with the goal of increasing the genetic diversity among the frogs bred in their facilities.

The practices of bullfrog farmers in Brazil tend to generate an undifferentiated population in the country over time. The same process seems to have occurred in other countries where populations were analyzed with the same genetic markers used in this study. Similar patterns were observed in China^43^—where also only two haplotypes were found among over 500 samples—and in some degree in Europe (5 haplotypes among nearly 400 samples from 8 countries)^47^, although European countries have more populations and more genetic diversity, due to the relatively high number of introduction events that happened in the continent (at least 25)^51^. The process of transforming introduced populations into a single population due to exchange of migrants may obscure population and invasion genetics inference, as the genetic signal is lost making it nearly impossible to clarify the history of events that followed the introductions. This also brings consequences to management efforts as important information like the origin and the dynamics of the introduced populations are let largely unanswered.

The number of introduction events seems to be directly related to the degree of genetic diversity in the region, and the bullfrog populations in Brazil exhibit the lowest number of mtDNA haplotypes of all studied non-native populations of this species examined so far^43,47,52^. Although the genetic diversity of bullfrogs in Brazil seems to be low, especially when compared to populations in its native range (42 haplotypes were found in the United States^79^), farming does not seem to suffer any impacts.

The exceptionally reduced genetic variation found in the population from Minas Gerais might explain the observation of morphological anomalies reported by Ferrante et al.^35^. Conversely, we observed a small percentage of anomalous animals when visiting farms, and farmers did not report any negative effects in their business. Research focused on other invasive amphibian species, such as the cane toad (*Rhinella marina* + *R. horribilis* species complex), show that reduction in genetic diversity after introduction does not seem to affect ecologically relevant traits^80^. This matter should be accounted when evaluating the potential of bullfrog invasiveness. Feral bullfrogs are highly associated to leaks from farms, and control measures should focus on preventing escapes using other resources than genetics, as feral and captive populations do not differ. The limited genetic variability present in the country is associated to the small number of introductions and founders.

## Supplementary Information

Supplementary Information 1.

Supplementary Information 2.

Supplementary Information 3.

## Data Availability

Available as Supplementary Data 1 and 2.
