# Supplementary figures and images for: Genetic structure of American bullfrog populations in Brazil
Source: Sci Rep. 2022 Jun 15;12:9927. doi: 10.1038/s41598-022-13870-2 (PMC9200760; doi:10.1038/s41598-022-13870-2)

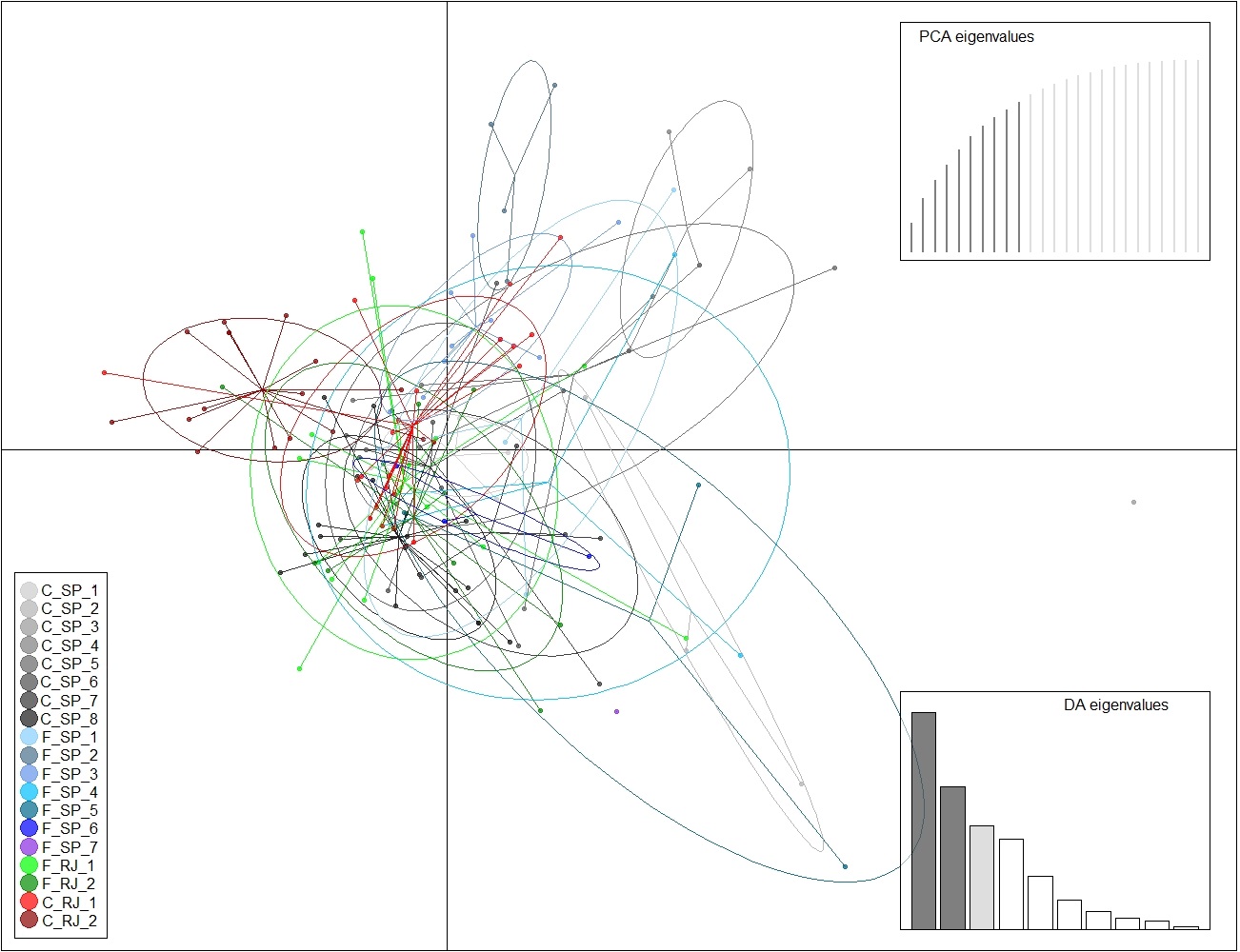

Supplement: Supplementary file 1 — Supplementary Information 1. [file 41598_2022_13870_MOESM1_ESM.jpeg]

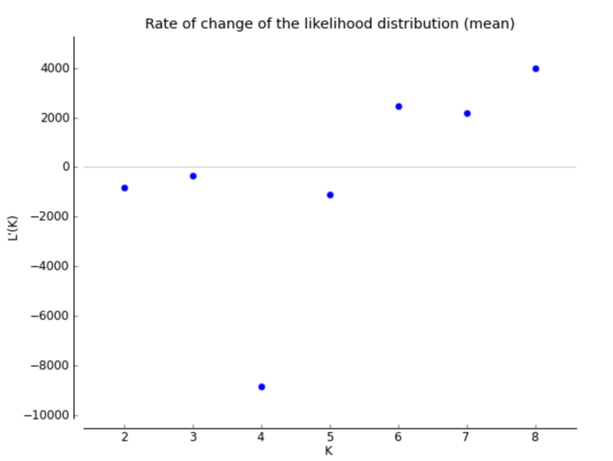

Supplement: Supplementary file 3 — Supplementary Information 3. [file 41598_2022_13870_MOESM3_ESM.png]
